# Retraction to “A data mining technique for detecting malignant mesothelioma cancer using multiple regression analysis”

**DOI:** 10.1515/biol-2023-0003

**Published:** 2024-12-19

**Authors:** Abdulla Mousa Falah Alali, Dhyaram Lakshmi Padmaja, Mukesh Soni, Muhammad Attique Khan, Faheem Khan, Isaac Ofori

**Affiliations:** Department of Computer Science, Isra University, Amman, Jordan; Department of Information Technology, Anurag University, Hyderabad, Telangana, India; Department of CSE, University Centre for Research & Development, Chandigarh University, Mohali, Punjab 140413, India; Department of Computer Science and Mathematics, Lebanese American University, Beirut, Lebanon; Department of Computer Engineering, Gachon University, Seongnam-si, South Korea; Department of Environmental and Safety Engineering, University of Mines and Technology (UMaT), Tarkwa, Ghana

Retraction of: Alali A, Padmaja D, Soni M, Khan M, Khan F, Ofori I. A data mining technique for detecting malignant mesothelioma cancer using multiple regression analysis. Open Life Sciences. 2023;18(1): 20220746. https://doi.org/10.1515/biol-2022-0746.

This article has been retracted by agreement between the Editor-in-Chief, Professor Thomas Litman, and the publisher, Walter De Gruyter GMBH, following a thorough investigation. The retraction is due to a suspected manipulation of authorship. Specifically, evidence indicates a possible misrepresentation of contributions by the listed authors, raising concerns about the legitimacy of the authorship claims and the integrity of the work.

